# Cuproptosis‐related molecular patterns and gene (ATP7A) in hepatocellular carcinoma and their relationships with tumor immune microenvironment and clinical features

**DOI:** 10.1002/cnr2.1904

**Published:** 2023-10-26

**Authors:** Shanbao Li, Junyong Weng, Chao Xiao, Jing Lu, Wanyue Cao, Fangbin Song, Zeping He, Peng Zhang, Zhonglin Zhu, Junming Xu

**Affiliations:** ^1^ Department of General Surgery, Shanghai General Hospital Shanghai Jiao Tong University School of Medicine Shanghai China; ^2^ Department of Colorectal Surgery Fudan University Shanghai Cancer Center Shanghai China; ^3^ Department of General Surgery Fudan University Huashan Hospital Shanghai China

**Keywords:** ATP7A, cuproptosis, hepatocellular carcinoma, immunotherapy, tumor microenvironment

## Abstract

**Background:**

Cuproptosis has been studied in various aspects as a new form of cell death.

**Aims:**

We hope to explore the molecular patterns and genes related to cuproptosis in evaluating and predicting the prognosis of hepatocellular carcinoma (HCC), as well as the impact of tumor immune microenvironment.

**Methods and Results:**

Sixteen cuproptosis related gene (CRGs) and cuproptosis related molecular and gene characteristics were comprehensively analyzed from 492 HCC samples. Cuproptosis related molecular patterns were generated by consensus clustering algorithm, including cuproptosis clusters, cuproptosis gene clusters (CGC) and cuproptosis score (CS). The characteristics of tumor microenvironment (TME) and tumor immune cells were described by the ssGSEA and ESTIMATE algorithms. Cuproptosis score was established to assess the clinical characteristics, prognostic and immunotherapy. The role and mechanism of CRG (ATP7A) in HCC, as well as its relationship with TME and immune checkpoints, have been further explored. The results of somatic mutation, copy number variations (CNV), and CRGs expression in HCC suggested the CRGs might participate in the HCC oncogenesis. The cuproptosis clusters were closely related to the clinical pathological characteristics, biological processes, and prognosis of HCC. The three CGC was revealed to be consistent with the three immune infiltration characterizations, including immune‐high, immune‐mid, and immune‐low subtypes. Higher CS was characterized by decreased TMB, activated immunity, higher immune cell proportion score (IPS) and better overall survival (OS), which indicated higher CS was immune‐high type and with better treatment effect and prognosis. The ATP7A had the highest hazard ratio (HR = 1.465, *p* < .001), was high expression in HCC tissues and with a shorter 5‐year OS. Knocking down ATP7A could enhance intracellular copper concentration, cause a decrease in DLAT expression, and induce cuproptosis and inhibit cell proliferation and migration. ATP7A was also positively correlated with most cancer immune cells and immune checkpoints.

**Conclusion:**

Taken together, this research revealed the cuproptosis related molecular patterns and genes associated with the clinical pathological characteristics, TME phenotype and prognosis of HCC. The CS will further deepen our understanding of the TME characteristics of HCC, and the involvement of ATP7A in cuproptosis will provide new ideas for predicting HCC prognosis and immunotherapy.

## 1 INTRODUCTION

Hepatocellular carcinoma (HCC) is a prevalent malignancy worldwide. It is also the fourth largest incidence rate and third largest cause of mortality in China.^1^, ^2^ Early stage of HCC could be treated curatively with surgery, but there is merely palliative treatment in advanced stages. The prognosis of HCC is poor because it is prone to vascular invasion and distant metastasis. However, the mechanism of HCC is unclear and requires in‐depth research.

“Cuproptosis” is a new kind of copper‐dependent cell death, the copper accumulation could induce the aggregation of mitochondrial lipoylated proteins and the instability of iron–sulfur cluster proteins, resulting in “Cuproptosis,” which is distinct from apoptosis, necroptosis, pyroptosis and ferroptosis.^3^ Copper is an important mineral nutrient, which is not only involved in mitochondrial respiration, iron uptake, and antioxidant/detoxification processes for all living organisms, but also as a factor in regulating or inducing some biological pathways in signal transduction including lipolysis, proliferation, and autophagy.^4^, ^5^ The copper transport proteins 1–5 (CTP1‐5) has extremely strong transport ability, especially the SLC31A1 (CTP1).^6^ P‐type ATPases (ATP7A, ATP7B) can mediate the excretion of copper, whose *N*‐terminal possesses metal binding sites.^7^ Dihydrolipoamide branched chain transacylase E2 (DBT), dihydrolipoamide S‐succinyltransferase (DLST), glycine cleavage system protein H (GCSH), dihydrolipoamide S‐Acetyltransferase (DLAT), pyruvate dehydrogenase (PDH) and ferredoxin 1(FDX1) are all involved in protein lipoylation, which in turn induce the cuproptosis and affects the TCA cycle. Moreover, cuproptosis mechanisms are shared by genetic models of copper homeostasis dysregulation.^3^ In several cancer types, copper levels in patients' blood and tissues have been found higher than normal people, such as pancreatic, gastric, lung and breast cancers.^5^, ^8^, ^9^ The homeostasis of copper plays an important role in cancers, and copper accumulation is related to tumor proliferation and metastasis.^6^, ^7^ The metabolism of copper in liver conditions and the development of HCC are still under research. The Wilson disease is caused by accumulation of copper in tissues based on the mutation in ATP7B, might enhance the incidence of HCC.^10^ Many studies have found that copper levels are closely related to liver cirrhosis and HCC.^11^, ^12^, ^13^ The above indicates the role of copper in the occurrence of HCC, indicating that cuproptosis may also be closely related to HCC. However, further research is needed on the correlation between cuproptosis and tumor promotion of copper accumulation, as well as how to utilize cuproptosis in clinical tumor treatment.

Tumor microenvironment (TME) is mainly composed of tumor cells and stromal components, which is closely associated with tumor progression, radiotherapy, chemotherapy and immunotherapy.^14^, ^15^ The residential fibroblasts, endothelial cells, infiltrating immune cells, secreted cytokines and chemokines, nascent blood and lymphatic vessels form the stromal components.^16^ Therefore, exploring the promoting or inhibiting behavior of TME on tumors and its involvement in the immune escape process of tumors is very complicated. The liver has immune defense and immune regulation functions with a complex liver immune microenvironment. The HCC parenchyma, stromal and infiltrating immune cells together constituted the immunosuppressive TME. In the TME of HCC, increasing the expression of immune checkpoint molecules on the cell surface enhances overall immunogenicity and promotes tumor immune escape.^17^, ^18^ The immune checkpoint inhibitors (ICIs), including anti‐PD1 or anti‐CTLA‐4 were widespread used in the second‐line therapy of HCC and other tumors.^19^, ^20^, ^21^ Additionally, lots of immune cells, infiltrating T cells, stromal cells and cancer‐activated fibroblast filled in blood and lymphatic vessels, which makes up a complex system of TME.^22^ According to the immune characteristics of TME of HCC and its relationship with molecular classification, the immunity of HCC is divided into the follow immune subtypes: high‐, mid‐, and low‐immune subtypes.^23^, ^24^, ^25^ The high‐immune subtype was characterized by increased plasma cells, and T lymphocytes infiltration, and other immune cell types also increased to varying degrees. The mid‐immune subtype was characterized by increased T lymphocytes and other immune cells infiltration, B and plasma cells infiltration were lesser. The less immune cells infiltration was low‐immune subtype. Recent research found that copper is closely correlated with the expression of hypoxia‐inducible factor 1α (HIF‐1α), which could stimulate angiogenesis, and neovascularization in turn induces the production of vascular endothelial growth factor (VEGF).^26^, ^27^ During the growth and metastasis of HCC, the vascular system and immune TME are influenced by VEGF.^28^, ^29^ Therefore, cuproptosis may play an important role in TME and immunotherapy in HCC. For instance, FDX1 was lower expression in HCC patients, which could induce HCC cell resistance to cuproptosis.^12^ The cancer associated fibroblasts (CAFs) as an important component of the TME, which not only secrete a variety of substances to participate in the regulation of the TME, but is also closely associated with the tumor metastasis, immunotherapy, and chemotherapy resistance.^30^ The arecoline could inhibit cuproptosis, significantly increasing the viability of CAFs in oral squamous cell carcinoma.^31^ However, the mechanism of cuproptosis in liver condition and the development of HCC is still unclear and further research is needed.

## 2 MATERIAL AND METHODS

### 2.1 Source and pretreatment of data set

The Figure S1 showed the workflow of this study. Public gene expression and clinical data of HCC profiles were obtained from the GEO and TCGA databases. GSE76427 and TCGA‐HCC cohorts were included. The samples we choose need to have complete pathological information and prognosis, including age, gender, TNM staging, survival time, and survival status. The RNA sequencing information of FPKM values were changed into TPM form in TCGA, including 374 cancers, while in GSE76427 was 115 cancers.^32^ 371 patients from the TCGA cohort and 111 patients from the GEO cohort (GSE76427) were obtained. The clinical documents of HCC profiles were shown (Table S1, S2). Combat algorithm was used to correct batch effects. The CNV and somatic mutation profiles were got from the UCSC xena and TCGA, respectively.

### 2.2 Consensus clustering analysis of CRGs

Sixteen cuproptosis related gene (CRGs) are included seven upregulators (LIPT1, LIAS, FDX1, DLD, DLAT, PDHA1, PDHB), three downregulators (GLS, MTF1, CDKN2A), three carriers (SLC31A1, ATP7A, ATP7B), three enzymes (DBT, GCSH, DLST).^3^, ^33^ The algorithm of consensus clustering could establish the clusters' numbers and determine their stability and the clustering was performed based on the criteria before reported.^34^ According to CRGs expression, the HCC samples were divided into three different molecular categories by the “ConsensusClusterPlus” and consensus unsupervised clustering analysis.

### 2.3 Gene set variation analysis

The c2.cp.kegg.v7.5.1 and c5.go.v7.5.1 hallmark gene sets were obtained from MSigDB database and performed by gene set variation analysis (GSVA) for further research.^35^ *p*‐value was adjusted (*p* < .05). The functional annotation for CRGs was used by the “clusterProfiler” and the FDR <0.05 (cutoff value).

### 2.4 Relationship among the cuproptosis molecular subgroups with clinical features of HCC

Three cuproptosis subgroups, clinicopathological characteristics and prognosis were identified by analyzed by the algorithm of consensus clustering. Moreover, the overall survival in three cuproptosis subgroups were detected and the survival curves were obtained by the “survival” and “survminer” R packages, respectively.

### 2.5 Differentially expressed genes were identified in different phenotypes of cuproptosis

We used the limma R package by empirical bayesian approach to obtain the cuproptosis related differentially expressed genes (DEGs) among the different subtypes (adjusted *p* < .05). The functions, enriched pathways and annotations of DEGs and CRGs were detected by the R package (clusterProfiler), the FDR <0.05(cutoff value).

### 2.6 The relationship of cuproptosis molecular patterns and TME cell infiltration

The ssGSEA and CIBERSORT algorithm was applied to quantify the relative abundance of immune cell infiltration in the TME of HCC.^36^ We used TIMER to analyze the correlation between ATP7A and immune cells and immune checkpoints associated with HCC.^37^

### 2.7 The CS was constructed to explore the cuproptosis related prognostic

The cuproptosis scores were computed to quantify the cuproptosis pattern of each HCC patient. Firstly, the DEGs identified from different cuproptosis clusters and normalized among all HCC patients, then found out the overlap genes. Secondly, the samples were divided into three geneclusters (CGC‐A, B and C) for further analysis by adopting the unsupervised clustering way for analyzing the overlap DEGs. Finally, the univariate Cox regression model was used to screen for genes with significant prognostic relevance. Principal component analysis (PCA) was employed to established cuproptosis relevant gene signature and selected the signature scores. The cuproptosis score was defined with principal compnonet 1 and 2 as follows: cuproptosis score = ∑(PC1i + PC2i). PC1i and PC2i stand for the score of two‐dimensional intersect gene, respectively.^34^ Bottom on the correlation between CS, TME scores and survival time, using the “survminer” to define the cut‐off point for each data set grouping.

### 2.8 Correlation of cuproptosis score with TMB mutation and immunotherapy in HCC

To detect the somatic mutation of HCC patients in high and low group of CS, the mutation annotation format was obtained by the “maftools.” The TMB score of each HCC patient in CS groups was detected. These immunotherapeutic targets for clinical treatment of HCC were summarized, including PD1, PDL1, CTLA‐4, VEGF, VECFR, KIT, PDGFR, HGFR, CDHR16 and RAF,^38^ and then the expression of these immunotherapeutic targets was calculated due to the CS. The therapeutic differences of chemotherapeutic drugs (anti‐PD‐1 and anti‐CTLA‐4 antibody) in HCC patients were researched in the two CS groups, the complete immunotherapy clinical annotations of HCC could be obtained from TICA database (https://tcia.at/home).

### 2.9 Tissue specimens and cell culture

We collected 24 primary HCC tissues from January 2020 to December 2021 in Shanghai General Hospital, these samples had no other treatment prior to surgery and are preserved in liquid nitrogen. This research was approved by the institutional research ethics committee of Shanghai General Hospital, the informed consents were obtained from the patients. The liver cell line Lo2 was generous gifts from Mr. Jing Lu and HCC cell lines Huh‐7, PLC, LM3, HepG2, MHCC‐97H were generous gifts from the Dr. Peng Zhang and Dr. Chao Xiao. These cell lines were tested by short tandem repeat authentication in 2021 and 2020 separately. They were cultured in DMEM medium (Invitrogen, USA), the 10% fetal bovine serum (Gibco, USA) and 1% penicillin streptomycin (Gibco, USA) were added, maintained in a humidified incubator at 37°C in 5% CO2.

### 2.10 Primers, shRNAs, and cell transfection

The qRT‐PCR primers:

**Table:** 

Target genes	Primers
ATP7A	Forward: 5′‐GATCGGTCAGCAAGTCACTTAGA‐3′
	Reverse: 5′‐GCGCTCCAGGAACATAGAAGAAT‐3′
GAPDH	Forward: 5′‐GTCTCCTCTGACTTCAACAGCG‐3′
	Reverse: 5′‐ACCACCCTGTTGCTGTAGCCAA‐3′
shRNA	shRNA1: CCATTCATGTACTAGCACTAT
	shRNA2: GCAAGGTGTTCAGCGAATTAA shRNA3: CCTCTTGGTATGGATTGTAAT
Scramble shRNA	CCTAAGGTTAAGTCGCCCTCG

LM3 cells with ATP7A shRNA and scramble plasmids transfection by lipo2000 (Invitrogen, USA) were cultivated in six‐well plates when they reached about 80%–90% confluence and incubation for 48 h for the subsequent experiments.

### 2.11 Quantitative RT‐PCR

Total RNA was obtained from tissues and cells by Trizol (Sigma, USA). cDNA synthesis was conducted by the synthesis kit (EnzyArtisan, China). The mRNA was quantified by S6 Universal SYBR qPCR mix (EnzyArtisan, China). GAPDH was as internal reference.

### 2.12 Scratch wound healing assay

Transfected LM3 cells by ATP7A shRNA and scramble plasmids were cultivated in six well plates until reaching about 80%–90% confluence. Then, scratched the cells in the middle of the well by a sterile 200 μL pipette tip. Photographs were captured at 0 and 36 h after wounding, the width was quantified and compared to baseline value. The experiment was repeated three times.

### 2.13 Transwell migration assay

Transfected LM3 cells were cultivated in the upper storey of the transwell (Corning, USA) with 200 μL of serum free media, while the lower chamber was added into 600ul 10% FBS containing medium. In the lower chamber with LM3 cells were fixed and stained with methanol and 0.1% crystal violet, respectively. Ten random fields were counted.

### 2.14 Cell proliferation

Transfected LM3 and control cells were cultivated in 96‐well plates with 3000 cells and 10% FBS containing medium per well. CCK‐8 reagent (10 μL/per well) was added after culturing cells for 24, 48, 72 and 96 h and incubated another 2 h. Then the OD value was measured at 450 nm by the standard microplate reader.

### 2.15 Measurement of intracellular copper

Transfected LM3 cells were seeded in 6‐cm plates containing medium with 10% fetal bovine serum. According to the manufacturer's instructions, after cell culture for 24 h, cells were collected and resuspended, and ultrasonically degradation to detect intracellular copper (Elabscience, Wuhan, China).

### 2.16 Immunoblot analysis

Proteins were extracted from LM3 cells, the protein transferred onto PVDF membranes (Cat No: CCGL52TP1, Millipore, Billerica, MA) after electrophoresis. Membranes were blocked in NcmBlot blocking buffer (Cat No: P30500, NCM Biotech, Suzhou, China) for 10 min, and then incubated with primary antibodies at 4°C overnight. The primary antibodies included anti‐DLAT antibody (Cat No: 13426‐1‐AP, 1:1000, Proteintech, USA), GAPDH monoclonal antibody (Cat No: 60004‐1‐Ig, 1:5000, Proteintech, USA). The secondary antibodies included goat anti‐rabbit IgG (Cat No: SA00001‐2, 1:5000, Proteintech, USA). Proteins were detected by ECL regent (Cat No: 180‐501, Tanon, Shanghai, China).

### 2.17 Statistical analyses

We are using R version 4.1.3 for statistical analyses and GraphPad Prism (version 8.0; GraphPad Software, USA) for basic study analyses. Student's *t* tests were applied to assess differences between two groups. All experiments were conducted independently and repeated three times. The *p*‐value <.05 was set as the Statistical significance.

## 3 RESULTS

### 3.1 Genetic variation and expression of CRGs in HCC

**FIGURE 1:**
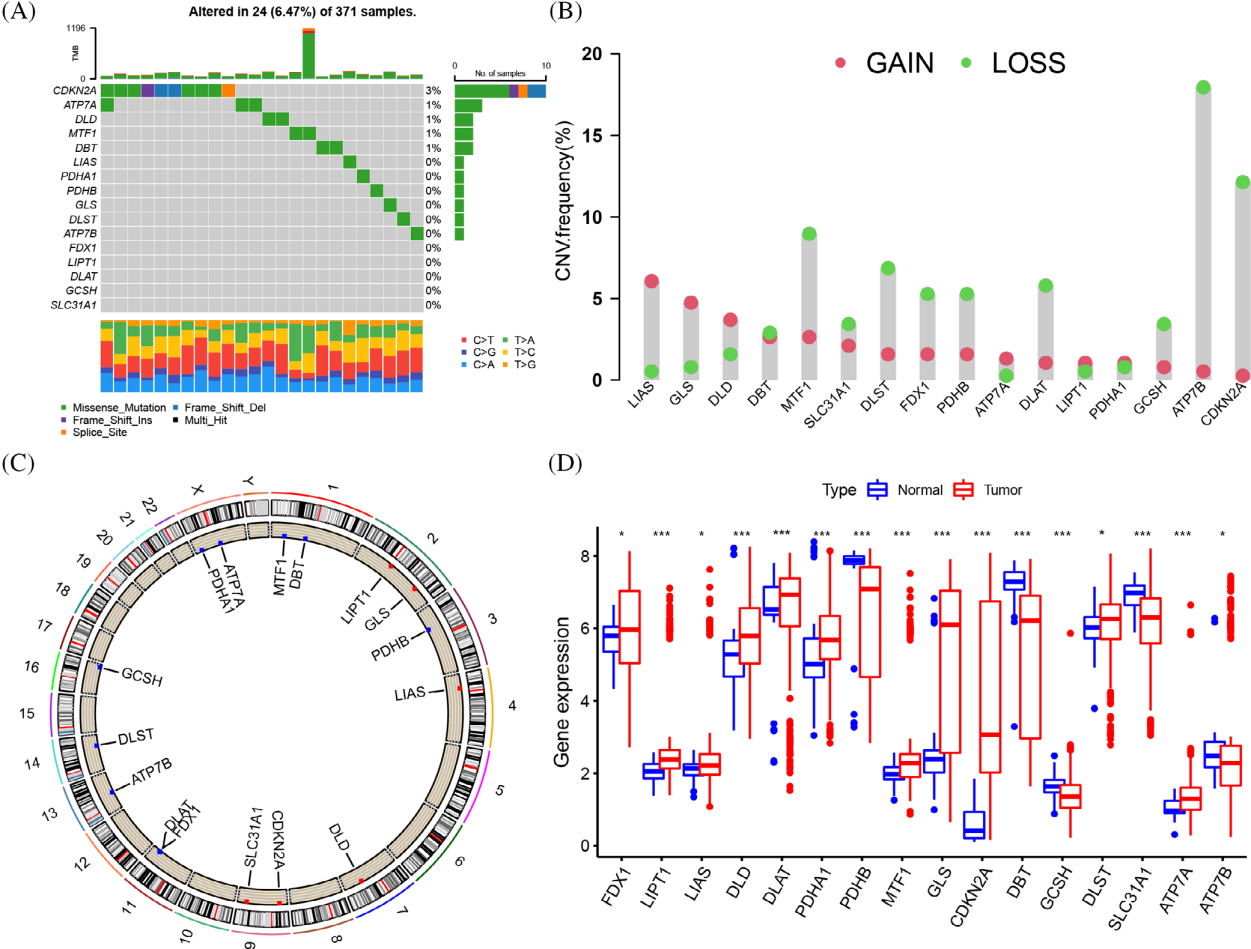
Genetic and transcriptional alterations of CRGs in HCC. (A) Mutation frequency of 16 CRGs in 371 HCC patients from the TCGA cohort. TMB is shown by the upper, sample fraction of conversions is shown by the below stacked bar plot, mutation frequency is shown by the right numbers and the bar plot indicates the proportion. (B) Variation frequencies of CNV among CRGs. The column height (alteration frequency), green dot (deletion frequency), red dot (amplification frequency). (C) Locations of CNV alterations of CRG regulators on 23 chromosomes. (D) The 16 CRGs expressed between normal tissues and HCC tissues. The “*” represented the statistical *p‐*value (*p < .05; **p < .01; ***p < .001).

The flowchart of this study was shown (Figure S1). The incidence of somatic mutations and copy number variations (CNV) were analyzed based on the 16 CRGs (Table S3). There were 24 experienced mutations (frequency 6.47%) in all HCC patients, while the CDKN2A gene showed the highest mutation frequency (Figure 1A). Further research has found that the GCSH significantly related to CDKN2A mutation status (Figure S2A,B). Simultaneously showing widespread CNV changes in all 16 CRGs, including an increase in LIAS, GLS, DLD, LIPT1, PDHA1 and ATP7A genes and a decrease in MTF1, DLST, FDX1, PDHB, DLAT, ATP7B, SLC31A1, GCSH and CDKN2A genes (Figure 1B). Moreover, the CNV alterations and locations were shown on chromosomes (Figure 1C). The mRNA expression of 16 CRGs was detected in normal and HCC samples, the expression of FDX1, LIAS, LIPT1, DLD, DLAT, PDHA1, MTF1, GLS, CDKN2A, DLST and ATP7A was higher in tumor, the expression of PDHB, DBT, GCSH, SLC31A1 and ATP7B was lower in tumor (Figure 1D). We observed similar CNV alterations and mRNA expression trends in most CRGs, indicating that CNV might affect the mRNA expression of CRGs (Figure 1B,D). Our results showed the genetic variation and expression of CRGs with a significant difference in HCC and normal samples, suggesting that the CRGs might participate in the HCC oncogenesis.

### 3.2 Identification of cuproptosis subtypes in HCC

**FIGURE 2:**
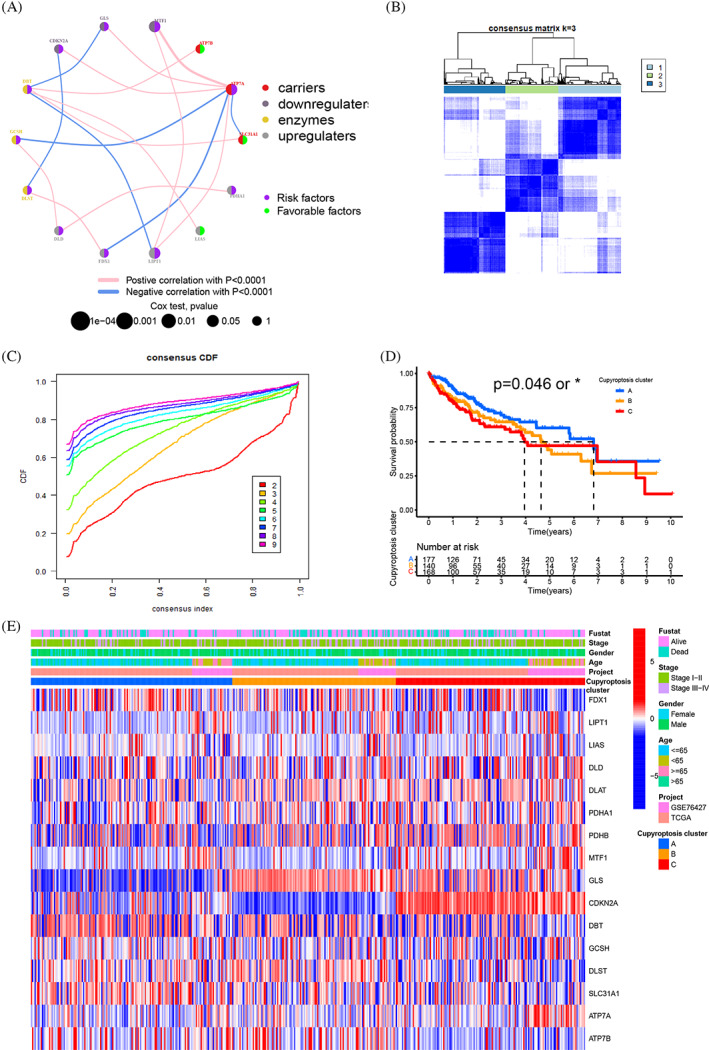
CRGs subtypes, clinicopathological, and biological characteristics of cuproptosis related patterns. (a) Interactions among 16 CRGs in HCC. The size of circle represented the effect of each CRGs on the prognosis, carriers (red dot), down‐regulators (dark green dot), up‐regulators (light green dot), enzymes (yellow dot), and the values calculated by Log‐rank test (*p* < .0001, *p* < .001, *p* < .01, *p* < .05 and *p* < 1). The lines linking CRGs showed their interactions, correlation strength between CRGs (line thickness), negative correlation (Blue line), positive correlation (pink line). Risk factors of prognosis (purple dot), protective factors of prognosis (green dot). (B, C) Consensus matrix heatmap defining k = 3 and their correlation area. (D) Survival analyses for the three subtypes were based on 491 patients with HCC from TCGA cohort and one GEO cohort (*p* = .048). (E) Differences in clinicopathologic features and expression levels of CRGs between the three distinct subtypes, the patient annotations included tumor stage, survival status, gender and age. The “*” represented the statistical *p‐*value (*p < .05).

Three hundred seventy‐one patients from the TCGA cohort and 111 patients from the GEO cohort (GSE76427) were integrated into this study for further research, as shown in Tables S1 and S2. The prognostic values of 16 CRGs were evaluated by the univariate Cox regression model (Figure S3A). The ATP7B, LIAS and SLC31A1 were expressed higher in HCC samples and with a longer 5‐year OS, while other CRGs were expressed lower and with a shorter 5‐year OS (Figure 1D, S3B–L, Table S4). The connection of 16 CRGs and their prognostic significance in HCC patients are shown in the cuproptosis landscape network, which suggested that these CRGs were significantly related to the prognosis of HCC (Figure 2A). Above indicated the CRGs were related to prognosis of HCC. Basing on the information of CRGs, the patients were categorized by the consensus clustering algorithm (Figure S4). The co‐expression clustering analysis indicated k = 3 is the best choice to divide into three subtypes, including cluster A (*n* = 206), B (*n* = 162) and C (*n* = 171) (Figure 2B,C) (Table S5). The Kaplan–Meier curves and clinical characteristic showed a longer 5‐year OS, higher alive status and lower stage with cuproptosis cluster A than cluster B and C (*p* = .048) (Figure 2D). In addition, significant differences were observed among the three cuproptosis clusters in terms of CRGs expression and clinical pathological features. Compared with cuproptosis cluster B and C, cluster A had a lower TNM staging (Figure 2E, Table S6).

### 3.3 The characteristics in different cuproptosis patterns

**FIGURE 3:**
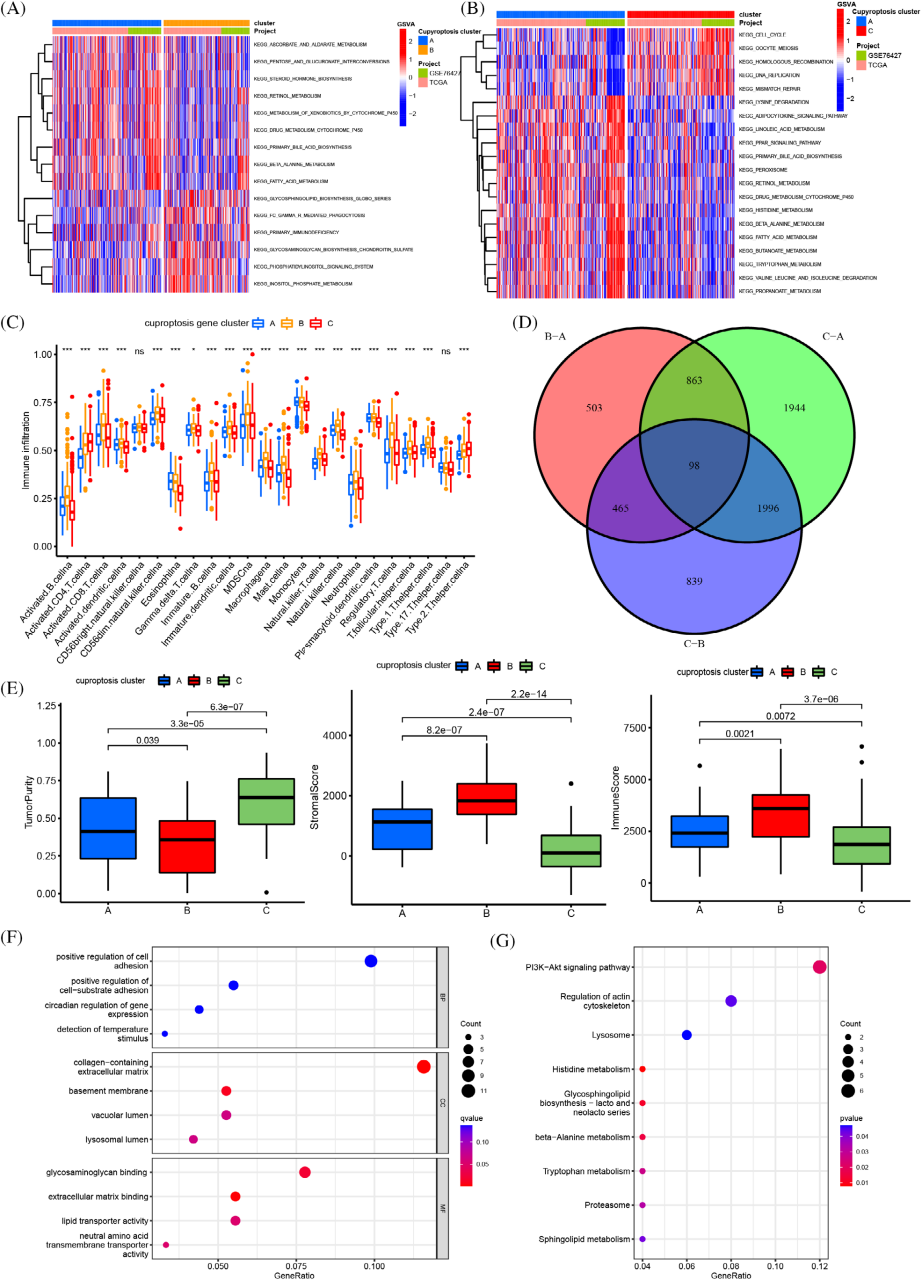
(A, B) GSVA enrichment analysis of biological pathways among three distinct cuproptosis subtypes, in which red represents activated pathways and blue represents inhibited pathways (A cluster A vs. cluster B, B cluster B vs. cluster C). (C) The abundance of each TME infiltrating immune cells in the three gene clusters, interquartile range of values (upper and lower ends of the boxes), median value (lines in the boxes), outliers (blue, red and yellow dots). (D) Differential genes of three gene clusters and 98 related genes shown in Venn diagram. (E) The stromal score, immune score and tumor purity in three cuproptosis subtypes by ESTIMATE algorithm. (F, G) GO and KEGG enrichment analyses of DEGs among three cuproptosis subtypes. (*p < .05; **p < .01; ***p < .001).

The three cuproptosis clusters were further analyzed by the GSVA. The cluster‐A was markedly enriched in metabolism, biosynthesis, and immunity, including drug metabolism cytochrome P450, tryptophan metabolism, PPAR signaling pathway, and primary immunodeficiency (Figure 3A). Furthermore, the tryptophan metabolism was involved in the tumor immunomodulatory process.^39^ PPAR signaling pathway is related to glucose metabolism and affected angiogenesis, and glycolysis is crucial for cancer cells, because glucose metabolism not only weakens their malignant potential, but also makes them sensitive to Cu ionophores therapy.^40^ All the above play a role in the TME of tumors. The cluster‐B was associated with metabolism and complement activation, such as complement and condensation cascade, and propionic acid metabolism. The cluster‐C was related to chromosome, such as RNA polymerase, DNA replication, and mismatch repair (Figure 3B, S5). The cluster‐A had the best 5‐year OS of HCC patients among the cuproptosis clusters, which indicated that the cluster‐A group might have immune activation effect on TME of HCC.

### 3.4 Identification of cuproptosis gene clusters and TME cell infiltration characteristics

**FIGURE 4:**
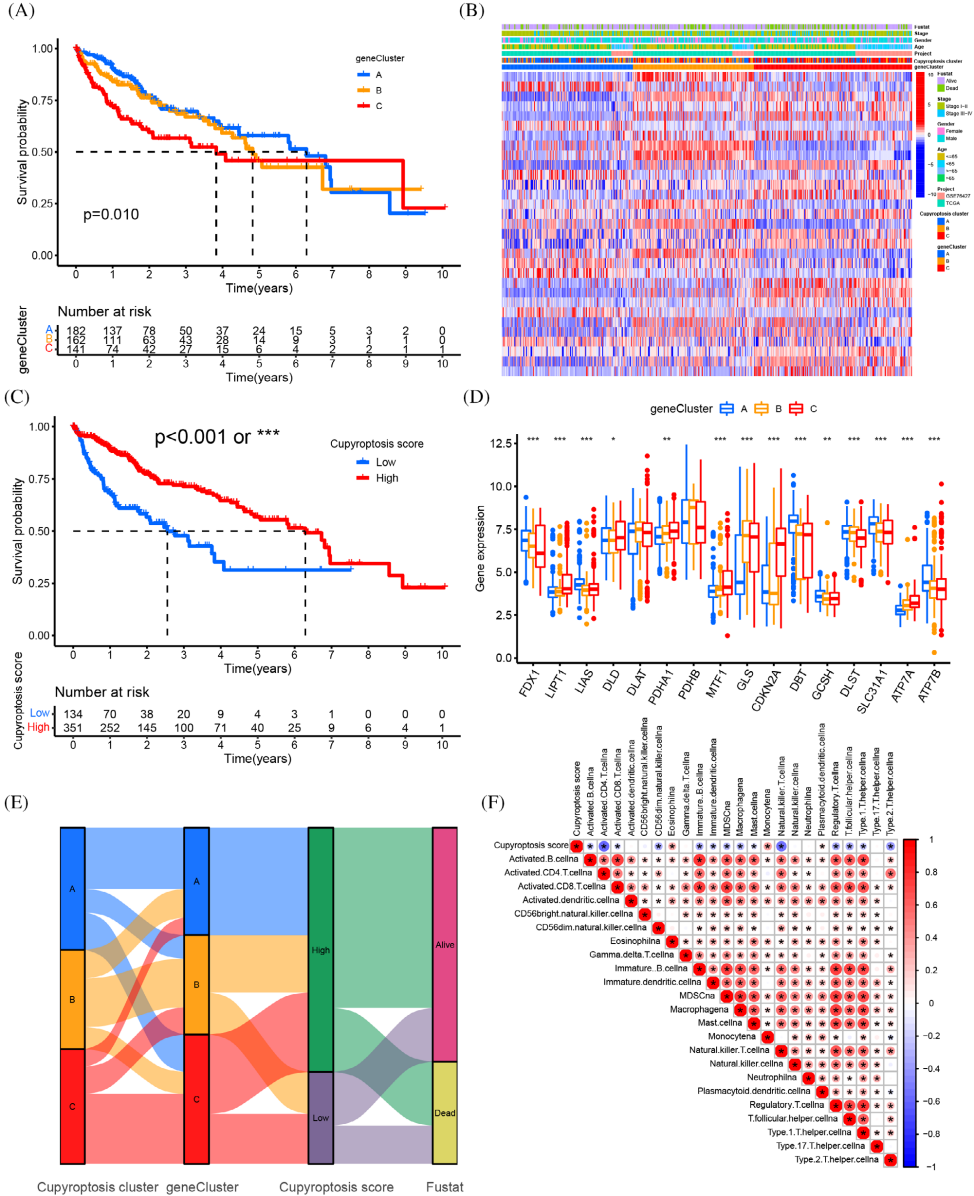
Construction of cuproptosis signatures. (A) Kaplan–Meier curves for OS of the three gene subtypes (*p* = .01). (B) Relationships between clinicopathologic features and the three gene subtypes. The patient annotations included gene clusters, cuproptosis clusters, tumor stage, survival status, gender and age. (C) Kaplan–Meier curves for RFS of the cuproptosis score subtypes (log‐rank tests, *p* < .001). (D) The expression of 16 CRGs in three gene cluster, interquartile range of values (upper and lower ends of the boxes), median value (lines in the boxes), outliers (blue, red and yellow dots). (E) Alluvial diagram of subgroup distributions in groups with different CRGs scores and survival outcomes. (F) Correlations between cuproptosis score and the known gene signatures by Spearman analysis. Negative correlation (blue) and positive correlation (red). The “*” represented the statistical *p‐*value (*p < .05; **p < .01; ***p < .001).

Cuproptosis gene clusters (CGC) was generated by the consensus clustering algorithm method, including CGC‐A, B and C (Figure S6). The correlation between the different CGC and 23 human immune cell subgroups of each HCC sample was evaluated by the ssGSEA to understand the role of CRGs in the TME of HCC samples. The ssGSEA results indicated the infiltration levels of B cells, T cells and plasma‐cells were obviously higher in CGC‐B, CGC‐A followed and CGC‐C was the lowest (Figure 3C) (Table S7). Combined with CGC‐A and B, both has better 5‐year OS, CGC‐C was the shorter, we attributed CGC‐B to the “Immune‐high subtype,” CGC‐A to “Immune‐mid subtype,” and CGC‐C to “Immune‐low subtype.” CGC‐C had the highest tumor purity and CGC‐B had the highest stromal and immune score, indicating CGC‐C possess more tumor parenchyma and CGC‐B more stromal cell components (Figure 3D,E). Cuproptosis cluster‐related DEGs were identified and conducted functional enrichment analysis to research the deep biological behavior of the cuproptosis patterns (Figure 3E). GO and KEGG analysis were performed and these DEGs were significantly correlated with positive regulation of cell adhesion, tryptophan metabolic and PI3K/AKT pathway, indicating the DEGs might be closely involved to the TME and immunotherapy of HCC (Figure 3F,G) (Table S8). Kaplan–Meier curves results indicated that patients with CGC‐A and B had the best 5‐year OS, while patients in CGC‐C showed the worst 5‐year OS (Figure 4A). CGC‐A and B groups were also associated with lower TNM stage than CGC‐C group and three CGC showed obvious differences in CRGs expression, consistent with the results of the cuproptosis molecular patterns (Figure 4B,D). Taken together, we identified the TME infiltration features in three distinct CGC.

### 3.5 The prognostic cuproptosis scores (CS) were constructed and validated

**FIGURE 5:**
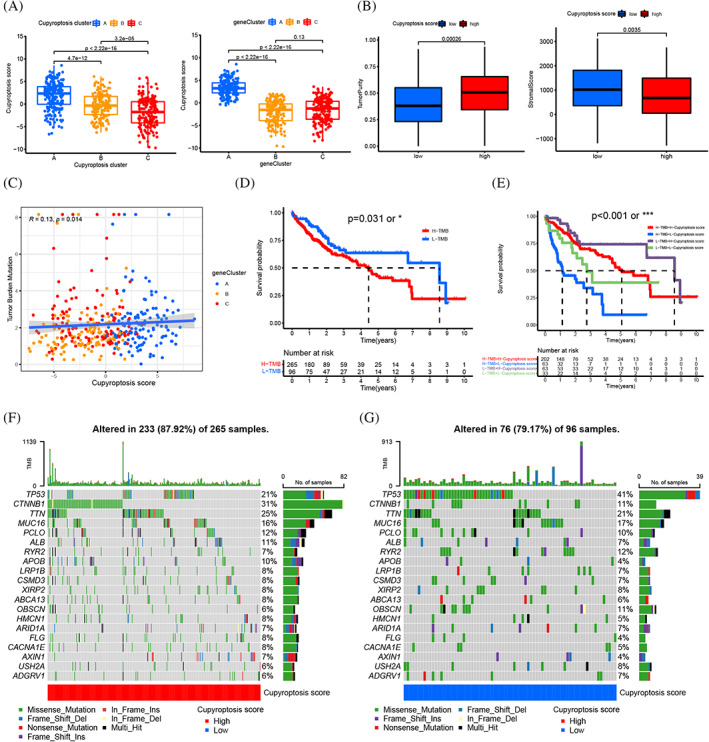
(A) Differences in cuproptosis scores between three gene subtypes and the three cuproptosis subtypes. (B) The stromal score and tumor purity in two cuproptosis scores by ESTIMATE algorithm. (C) Spearman correlation analysis of the cuproptosis score and TMB. (D) Survival analyses for low and high TMB score patient groups using Kaplan–Meier curves (*p* < .031). (C) Survival analyses for subgroup patients stratified by both cuproptosis and TMB score by Kaplan–Meier curves. H, high; L, Low (*p* < .001). (F, G) The waterfall plot of somatic mutation features established with high and low cuproptosis scores. TMB (The upper barplot), mutation frequency in each gene (the number on the right), proportion of each variant type (right barplot). The “*” represented the statistical *p‐*value (*p < .05; **p < .01; ***p < .001).

**FIGURE 6:**
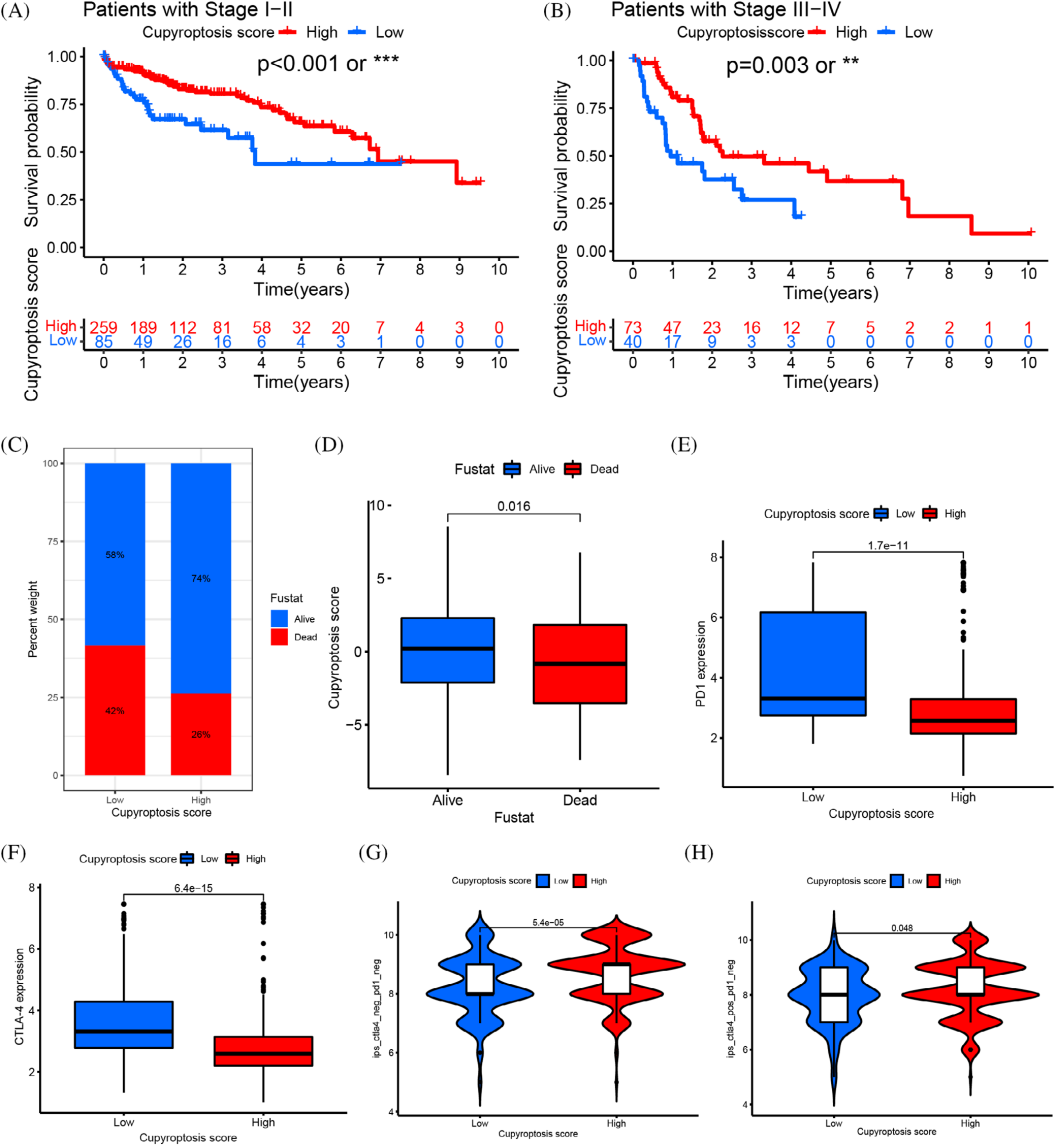
(A, B) Survival analysis of low and high cuproptosis score patient groups in tumor stages I, II and stages III, IV by Log‐rank test. A (stages I, II, *p* < .001) and B (stages III. IV, *p* = .003). (C) The proportion of patient survival state in low or high cuproptosis score groups. (D) Distribution of cuproptosis score in alive and dead groups (*p* = .016). (E) Differences in PD‐1 expression between low and high cuproptosis score groups (*p* < .001). (F) Differences in CTLA‐4 expression between low and high cuproptosis score groups (*p* < .0001). (G) The patients with response to PD‐1 and CTLA‐4 blockade immunotherapy in low or high cuproptosis score groups. The high group had positive response to CTLA‐4 treatment (*p* = .048). The “*” represented the statistical *p‐*value (*p < .05; **p < .01; ***p < .001).

The CS were established due to the subtype related DEGs (Table S9). The distribution of HCC patients in cuproptosis clusters, three CGC, and CS was shown (Figure 4E). The correlation between CS and immune cells showed the CS was closely related to monocytes, activated CD4 T cells, CD56 NK cells, natural killer T cells, type1/2 helper T cells, immature B cells, MDSCs, macrophages and regular T cells (Figure 4F). The significant differences in CS between CGS were observed by Kruskal Wallis test. The score of CGC‐A was the highest, CGC‐C was the second and CGC‐B was the lowest, which indicated that the score of CGC‐A might be closely related to stromal immune activation characteristics (Figure 5A). More important, cuproptosis cluster‐A showed the significantly increased CS, while cuproptosis cluster‐B and C presented no difference (Figure 5A). Next, samples with low CS showed higher stromal score and lower tumor purity, implying immune high phenotype; the high CS showed lower stromal score and higher tumor purity, implying immune low phenotype (Figure 5B). The survival curves showed the patients with high CS had a positive OS of HCC patients in stages I and II (*p* < .001) and III‐IV(*p* = .003), respectively (Figure 6A,B). Based on the analysis of the future survival status of HCC patients, it was found that the living patients in high group of CS were better than those in low group (Figure 6C,D). Our results revealed that high CS group performed a significantly better prognosis.

### 3.6 Evaluation of TMB characterization and immune checkpoints between the cuproptosis scores

The mutation was analyzed by spearman correlation analysis and results showed CS was closely related to the TMB (Figure 5C). Next, we observed the distribution variations of somatic mutations between the two CS groups. The HCC patients with high TMB had a worse OS (Figure 5D). Survival analysis of four HCC patient groups stratified by both CS and TMB was conducted, we found the low CS with high TMB showed a poor OS and high CS group with low TMB showed a much better survival advantage (Figure 5E). The top eight genes of mutation in the CS groups were TTN, ALB, RYR2, TP53, CTNNB1, MUC16, PCLO and APOB. The CTNNB1, TTN, PCLO, APOB and ALB had higher frequencies in high CS group, while the mutation levels of TP53, MUC16 and RYR2 were opposite (Figure 5F,G). To further research the connection between the CS and immunotherapy, we summarized the targets of immune related therapy for HCC, including PD1, PDL1, CTLA‐4, VEGF, VEGFR, KIT, PDGFR, HGFR, CDHR and RAF. Differences in immunotherapy target expression between CS groups in HCC were analyzed, these immunotherapy targets expressed lower in high CS group compared with the low CS group, such as PD1 (*p* < .001), PDL1 (*p* = .0037), CTLA‐4 (*p* < .001), KIT (*p* < .001); while the VEGF (*p* < .001), VEGFR (*p* = .0013), PDGFR (*p* = .043), HGFR (*p* = .027) and RAF (*p* < .001) (Figure 6E,F and S7A–H). In addition, the expression of GDHR16 with no statistically significant (Figure S7F). The existing immunotherapy data were obtained from the cancer immunome atlas. The response to PD‐1 and CTLA‐4 blockade immunotherapy in CS groups of HCC patients was assessed (Table S10). We found that the high CS group was positive response to CTLA‐4 (+)/PD1 (−) and CTLA‐4 (−)/PD1 (−) (Figure 6G). Our results indicated the CS was a latent biomarker for assessing the prognosis and immunotherapy response of HCC patients.

### 3.7 ATP7A was high expressed in HCC and predict the prognosis

**FIGURE 7:**
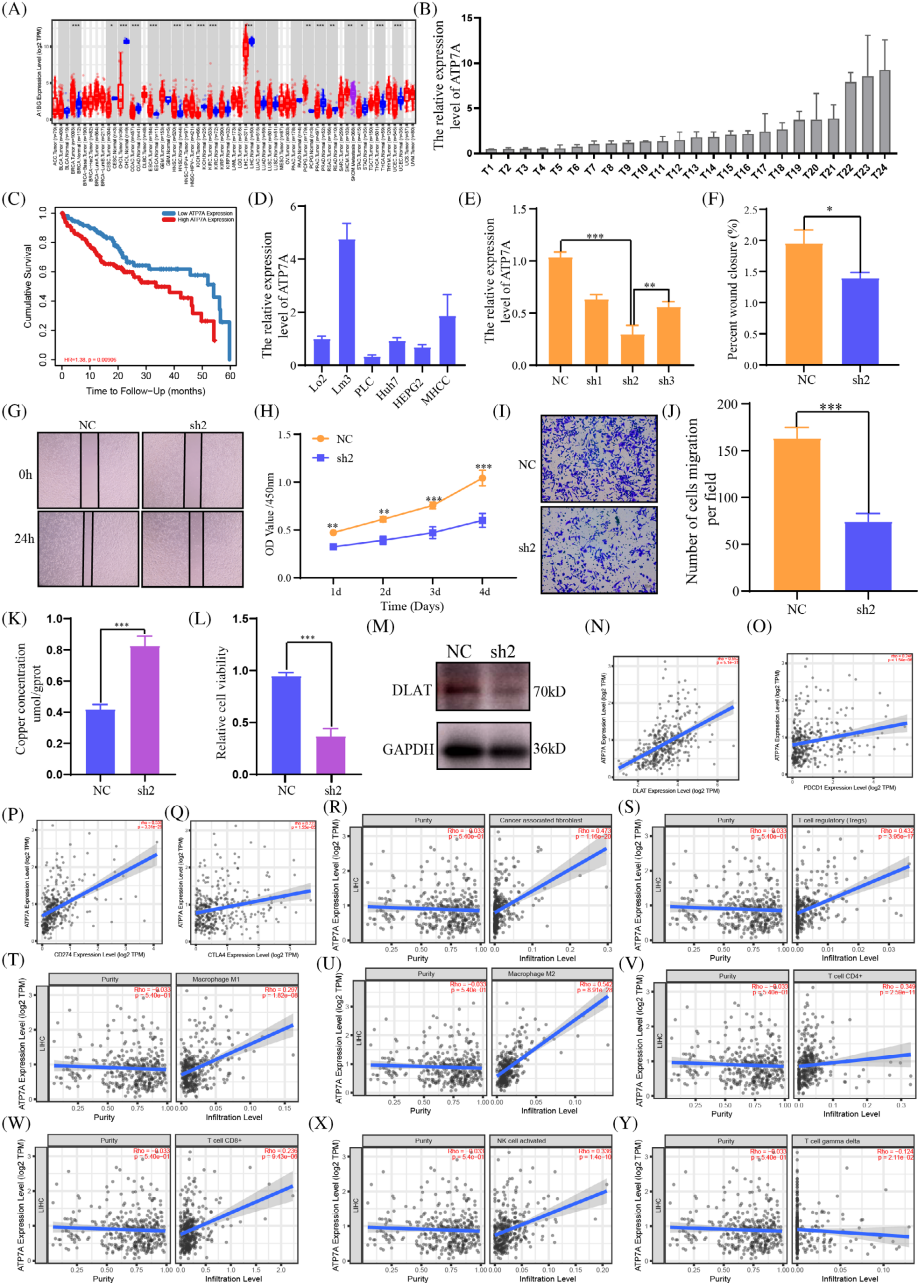
(A) The mRNA expression level of ATP7A in pan‐cancer. (B) The relative mRNA expression of ATP7A in 24 HCC tissues and paired normal adjacent tissues by qPCR. (C) The 5‐year OS between high and low expression of ATP7A in TCGA database by TIMER. (D) The relative mRNA expression of ATP7A in lo2 normal liver cell line and other five HCC cell lines. (E) LM3 cells were transfected with ATP7A silencing vectors and knockdown efficiency was verified by qPCR. (F, G) The effects of ATP7A knockdown on wound healing, (H) Cell proliferation, and (I, J) migration abilities of the indicated cell lines. (K) The concentration of copper was detected after ATP7A knockdown in LM3 cells. (L) Cell viability was detected after ATP7A knockdown in LM3 cells. (M) Representative marker DLAT of cuproptosis was detected by WB after ATP7A knockdown in LM3 cells. (N) The correlation between the expression of ATP7A and DLAT in HCC samples. The correlation between the expression of ATP7A and immune checkpoints, including (O) PD1, (P) PDL1 and (Q) CTLA4. The correlation between the expression of ATP7A and immune cells infiltration level in HCC by TIMER, including (R) cancer‐associated fibroblast, (S) Tregs, (T) macrophages M1, (U) macrophages M2, (V) CD4+ T cells, (W) CD8+ T cells, (X) NK cells and (Y) T cell gamma delta. Error bars represent the SEM. *p < .05, **p < .01 and ***p < .001 by two‐tailed Student's *t* test. Original magnification: ×100. NC group infected with negative control lentivirus, sh2 group infected with shRNA‐2.

The univariate Cox regression model and hazard ratio were used and the ATP7A got the highest score (HR = 1.465, *p* < .001) (Figure S3A). The mRNA expression level of ATP7A in pan‐cancer (Figure 7A). Next, we detected the ATP7A mRNA relative expression in 24 paired normal and HCC tissues by qRT‐PCR, the ATP7A was high expressed in most HCC tissues (Figure 7B). The high expression of ATP7A was with a shorter 5‐year OS (Figure 7C, S3F).

### 3.8 Low expression of ATP7A increased HCC cell copper accumulation and suppressed the proliferation and migration abilities of HCC cells

ATP7A mRNA expression were detected in different HCC cell lines. The result showed the expression of ATP7A mRNA was the highest in the LM3 (Figure 7D). The shRNA plasmids were used to knockdown ATP7A expression in LM3 cells and the sh2 sequence was verified the best knockdown efficiency by qRT‐PCR (Figure 7E). The wound healing and transwell migration assays indicated cells with lower ATP7A expression could suppress the proliferation and migration ability of LM3 cells, CCK‐8 assays showed the ATP7A knockdown evidently inhibited cell proliferation of LM3 (Figure 7F–J). We further evaluated whether knockdown of ATP7A led to changes in intracellular copper accumulation. The copper level increased after ATP7A knockdown (Figure 7K), while the trend of cell viability was opposite (Figure 7L), which suggested knockdown of ATP7A could induce increase in copper accumulation in HCC cells and inhibit HCC cells growth. Copper can bind to thioacylated DLAT and increase insoluble DLAT, leading to cytotoxicity and inducing cell death.^41^ We verified and found that after knocking down ATP7A, the expression of DLAT decreased in HCC cells (Figure 7M).

We further validated the positive correlation between ATP7A and DLAT in HCC samples, as well as immune checkpoints PD1, PDL1, and CTLA4 (Figure 7N–Q). We also investigated the relationship between ATP7A and tumor related immune cells and found that ATP7A is positively correlated with cancer associated fibroblast, Tregs, macrophages M1, macrophages M2, CD4^+^ T cells, CD8^+^ T cells and NK cells, but negatively correlated with T cell gamma delta. Collectively, low expression of ATP7A could induce cuproptosis and inhibit cell proliferation by regulating the increase of copper concentration in HCC cells; Simultaneously participating in the TME of HCC, regulating the expression of immune checkpoints in tumor immune related cells, and affecting tumor progression.

## 4 DISCUSSION

Cuproptosis is a novel cell death and considered the indispensable role in tumor development and progression.^40^ Related copper chelators and anti‐tumor drugs using copper carriers have been continuously discovered and tested in clinical trials. However, the correlation between CRGs and molecular patterns, clinicopathological characteristics, prognostic value, TME infiltration characteristics, and immunotherapy responses in HCC are unclear and still needs to be studied.^42^, ^43^

In this study, we screened out 16 CRGs and evaluated their somatic mutations, CNV, mRNA expression, and biological functions in HCC. We found 24 experienced mutations in all HCC patients and CDKN2A gene showed the highest mutation frequency. Previous studies have suggested that the enrichment of somatic mutations depends on the etiology and risk factor in HCC.^44^ The HBV induced HCC had a higher frequency of somatic mutations (TP53, CTNNB1) in apoptosis and ALD‐induced HCC was associated with CTNNB1, HGF, and CDKN2A mutations.^44^, ^45^ CNV is an important source of human genome variation, and also an important cause of tumor.^33^, ^46^ We observed similar CNV alterations and mRNA expression trends in most CRGs, indicating that CNV might affect the expression of CRGs. The 16 CRGs are closely related to copper homeostasis, protein thioacylation, TCA cycle, and oxidative stress responses. Moreover, most of these CRGs are the prognostic effectors of HCC patients, such as ATP7B, CDKN2A, DLAT and so forth.^47^, ^48^ The enrichment of CNV and somatic mutations related to cuproptosis suggested that cuproptosis may induce the occurrence of HCC.

We further identified cuproptosis clusters, CRGC, and CS molecular patterns, the results revealed overall alterations of CRGs in HCC. The cuproptosis cluster A had a longer 5‐year OS, higher alive status and lower TNM stage than cluster B and C. We found the cuproptosis cluster‐A was markedly enriched in metabolism, biosynthesis, and immunity, including drug metabolism cytochrome P450, tryptophan metabolism, PPAR signaling pathway, and primary immunodeficiency, all of these were involved in the TME of tumors. The tryptophan metabolism was involved in the tumor immunomodulatory process.^39^ PPAR signaling pathway is related to glucose metabolism and affected angiogenesis, and glycolysis is crucial for cancer cells, because glucose metabolism not only weakens their malignant potential, but also makes them sensitive to Cu ionophores therapy.^40^ We further discovered that CGC‐B had a better 5‐year OS and CGC‐C was the shortest. CGC‐C had the highest tumor purity and CGC‐B had the highest stromal and immune score, indicating CGC‐C possess more tumor parenchyma and CGC‐B more stromal cell components. The ssGSEA results indicated the CGC‐B contained activated CD4 T cells, activated CD8 T cells, NK cells, macrophage and Tregs, while the CGC‐C was only enriched in Th17 and Th2 cells. Activated CD8 T cells is involved in anti‐tumor immunity, CD8 T cell exhaustion affects anti‐tumor effects and the mechanism is complex,^49^ early‐relapse HCC have decreased levels of Tregs, increased DCs and infiltrated CD8^+^ T cells, exhausted CD8^+^ T cells have become the focus of anti‐tumor immunity research.^50^ Intra‐tumoral dendritic cell‐CD4^+^ T cell niches enable CD8^+^T cell differentiation following PD‐1 blockade in HCC.^51^ NK cells also have anti‐tumor effects, which are related to immunotherapy, TGF‐β could restore NK cell immunity against tumors by reversing NK cell exhaustion.^52^ Tregs and neutrophil extracellular trap interaction could contribute to carcinogenesis in non‐alcoholic steatohepatitis.^53^ The phenotype and functions of macrophages are induced by the surrounding microenvironment, which contain two classic subtypes M1 (proinflammatory and antitumor) and M2 (anti‐inflammatory and immunosuppressive) type macrophages. Macrophages can be induced to polarize by Th2 secreting cytokines IL‐4 and IL‐13, producing IL‐10 and TGF‐β anti‐inflammatory cytokines.^54^ Therefore, the CGC‐B attributed to the “Immune‐high subtype” and CGC‐C attributed to “Immune‐low subtype.” We demonstrated that the three CGC were characterized by distinct TME characteristics, which was also closely associated with the clinicopathological staging and OS. The novel molecular pattern was proved to be a reliable and effective classification of HCC patients and immune phenotype.

In view of cuproptosis plays an important role in the TME process of HCC, we constructed a CS model to predict the prognosis of individual samples and immune subtype. The low CS indicated the “Immune‐high subtype” with longer OS, while the high CS indicated the “Immune‐low subtype” with shorter OS. Research has shown that patients with high TMB are more likely to benefit from ICIs therapy.^55^ Our analysis showed the TMB was positively correlated with CS, the OS with high TMB was worse than low TMB and the low CS group, high TMB patients were with the worst OS compared with the low TMB and high CS group, indicating the CS and TMB might have synergistic effects in HCC. However, TMB is usually lower in HCC and there is no available data to support that TMB could predict immune reaction to ICIs.^56^

The use of ICIs for the treatment of HCC is widely used, especially the immune checkpoint PD1, PDL1 and CTLA4.^17^, ^21^ We also evaluated that the expression of PD1, CTLA4, PDL1, KIT and CDHR was lower in high CS group, while the VEGF, VEGFR, RAF, PDGFR and HGFR was higher. Moreover, using the existing immunotherapy data about PD1 and CTLA‐4 of HCC in the database, showing the immunotherapy of high CS group was positive response to CTLA‐4(+)/PD1(−) and CTLA‐4(−)/PD1(−). These results indicated the cuproptosis made a pivotal contribution to the TME and immunotherapy of HCC.

The univariate Cox proportional hazards regression model and risk results showed that ATP7A had the highest hazard ratio among those examined and high expression of ATP7A was with a shorter 5‐year OS, which suggested that ATP7A has a promoting effect on HCC progression. The ATP7A is an important component of intracellular copper homeostasis, the mutation of ATP7A is closely related to copper transport disorders,^57^ also involved in the progression of tumors.^8^ However, the role of ATP7A in HCC through cuproptosis is not very clear, and its impact on the immune microenvironment of HCC is also unknown. We verified the ATP7A was overexpressed in 24 pair of HCC tissues compared to adjacent normal tissues, and ATP7A knocked down could inhibit the HCC cells proliferation and migration. There is a positive correlation between ATP7A and DLAT expression in HCC. Copper could bind to thioacylated DLAT, inducing the isomerization of DLAT. The increase in insoluble DLAT leads to cytotoxicity and induces cell death.^41^ Knocking down of ATP7A could induce an increase in copper accumulation in HCC cells, decrease the expression of DLAT, and inhibit the HCC cells growth. Moreover, there is not only a positive correlation between ATP7A and the expression of immune checkpoints, such as PD1, PDL1, and CTLA4, but is also closely associated with CD4^+^ T cells, CD8^+^ T cells, Tregs, M1/M2 macrophages and NK cells. As mentioned above, ATP7A plays a crucial role in the immune TME and immune checkpoint regulatory of HCC by regulating the cuproptosis process, but the specific mechanism still needs further research.

Although we reveal the clinicopathological characteristics, TME phenotype, and vitro research of cuproptosis patterns in HCC, there are several shortcomings in this research. First, most data were obtained from public data set, and selection bias of samples may exist. Large sample prospective studies are still needed to verify these results. Second, we lack in vivo validation of the screened CRGs and validation after the interaction of immune cells and tumor cells in patients. Last, further research is needed on the specific mechanism of cuproptosis regulating the proliferation and metastasis of HCC and its role in TME.

## 5 CONCLUSION

This research revealed the cuproptosis‐related molecular patterns and genes associated with the clinical pathological characteristics, TME phenotype and prognosis of HCC. The CS will further deepen our understanding of the TME characteristics of HCC, and the involvement of ATP7A in cuproptosis will provide new ideas for predicting HCC prognosis and immunotherapy.

## AUTHOR CONTRIBUTIONS

**Shanbao Li:** Formal analysis (lead); methodology (lead); software (lead); supervision (lead); validation (lead); writing – original draft (lead). **Junyong Weng:** Formal analysis (equal); funding acquisition (supporting); writing – review and editing (equal). **Chao Xiao:** Conceptualization (equal); resources (lead). **Jing Lu:** Methodology (equal); software (equal). **Wanyue Cao:** Resources (equal); supervision (equal). **Fangbin Song:** Data curation (equal); formal analysis (equal); writing – review and editing (equal). **Zeping He:** Software (equal); supervision (equal). **Peng Zhang:** Resources (equal). **Zhonglin Zhu:** Conceptualization (lead); methodology (equal); writing – review and editing (lead). **Junming Xu:** Conceptualization (lead); funding acquisition (supporting); project administration (lead); resources (lead).

## FUNDING INFORMATION

This research was supported by the National Natural Science Foundation of China (8 197 032 698 and 82 003 060).

## CONFLICT OF INTEREST STATEMENT

The authors declare that they have no competing interests.

## ETHICS STATEMENT

This research was approved by the institutional research ethics committee of Shanghai General Hospital, the informed consents were obtained from the patients.

## Supporting information


**Figure S1.** The entire analytical process of the study.

**Figure S2.** The relationship between CDKN2A mutation and expression level of cuproptosis‐related genes in HCC.

**Figure S3. A**. The prognostic analyses for 16 CRGs in the two HCC cohorts using a univariate Cox regression model. Hazard ratio (>1) represented risk factors for survival and hazard ratio (<1) represented protective factors for survival. Survival analyses for low and high CRGs expression patient groups using Kaplan–Meier curves by Log‐rank test. **B** (CDKN2A, *P* = .019), **C** (DLAT, *P* < .001), **D** (FDX1, *P* = .020), **E** (GCSH, *P* = .012), **F** (ATP7A, *P* = 0.017), **G** (LIAS, *P* = .031), **H** (LIPT1, *P* = .006), **I** (MTF1, *P* = .001), **J** (PDHA1, *P* = .010), **K** (SLC31A1, *P* = .027), **L** (ATP7B, *P* = .013).

**Figure S4.** Unsupervised clustering of CRGs and Consensus matrix heatmaps for K (K = 2, 4, 5, 6, 7).

**Figure S5.** GSVA enrichment analysis of biological pathways between three distinct subtypes by KEGG (**A**, cluster A vs. cluster C) and Go (**B** cluster A vs. cluster B, C cluster A vs. cluster C, **C** cluster B vs. cluster C) analyses.

**Figure S6.** Identification of gene subtypes based on DEGs among three cuproptosis subtypes in HCC cohort.

**Figure S7.** Differences in immunotherapy target expression between low and high cuproptosis score groups in HCC. **A** (VECFR, *P* = .0013), **B** (PDL1, *P* = .0037), **C** (KIT, *P* < .001), **D** (PDGFR, *P* = .043), **E** (HGFR, *P* = .027), **F** (CDHR16, *P* = .38), **G** (VEGF, *P* < .001), **H** (RAF, *P* < .001).

**Table S1.** Clinical information of 377 LIHC patients from TCGA(unelection).
**Table S2:** Clinical information of 111 LIHC patients from GSE76427(after election).
**Table S3:** Summary of 16 recognized cuproptosis‐related genes.
**Table S4:** Survival status and OS of LIHC patients (TGCA/GSE76427).
**Table S5:** Cuproptosis cluster.
**Table S6:** Expression characteristics of CRGs.
**Table S7:** GO and KEGG enrichment analyses of DEGs among three cuproptosis subtypes.
**Table S8:** Relative Fractions of tumor‐infiltrating immune cells of LIHC patients.
**Table S9:** Cuproptosis score.
**Table S10:** Immunotherapy in LIHC patients(TICA datebase).

## Data Availability

The data used for the study are available from the corresponding and first author.
