# Supplementary figures and images for: Cuproptosis‐related molecular patterns and gene (ATP7A) in hepatocellular carcinoma and their relationships with tumor immune microenvironment and clinical features
Source: Cancer Rep (Hoboken). 2023 Oct 26;6(12):e1904. doi: 10.1002/cnr2.1904 (PMC10728522; doi:10.1002/cnr2.1904)

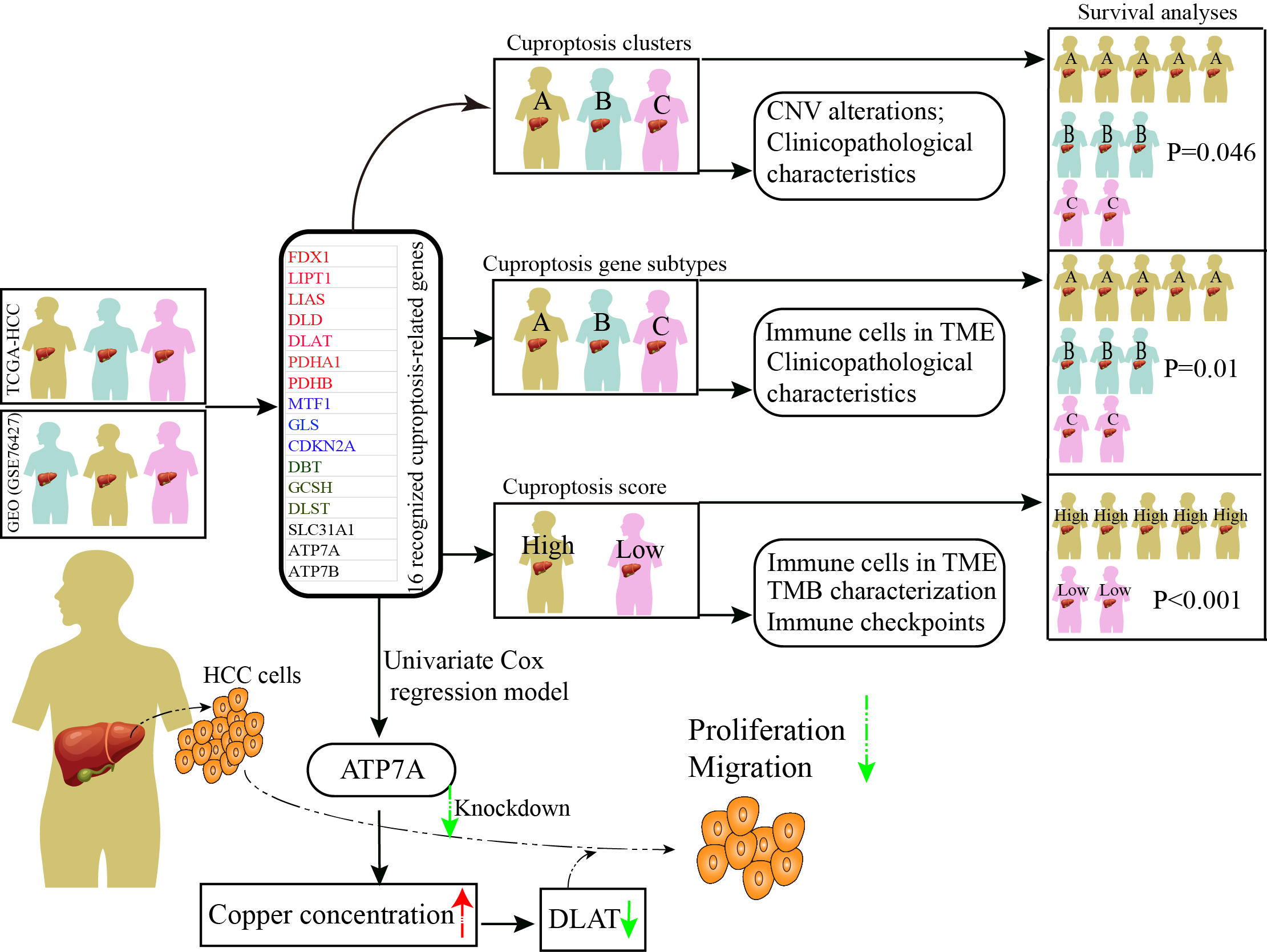

Supplement: Supplementary file 1 — Figure S1. The entire analytical process of the study. [file CNR2-6-e1904-s008.tif]

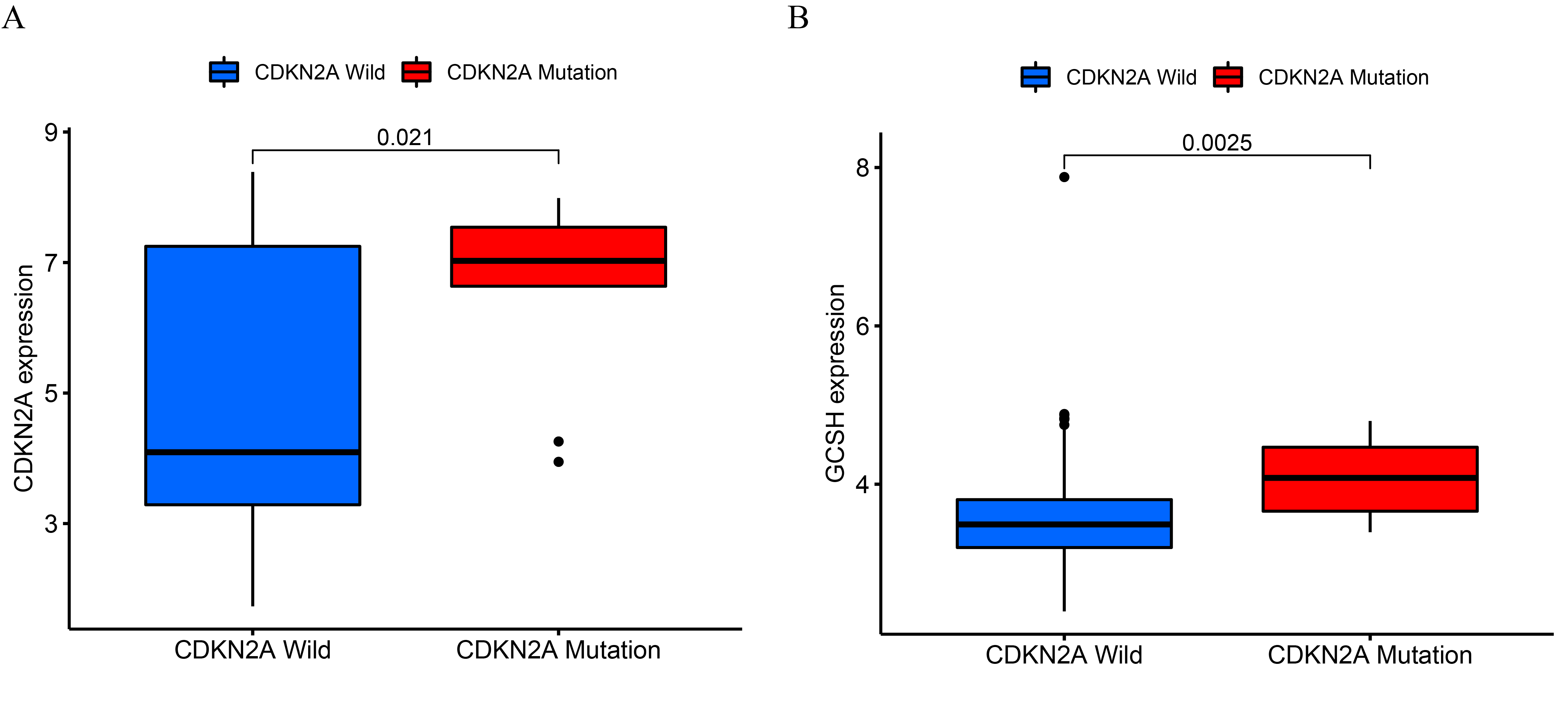

Supplement: Supplementary file 2 — Figure S2. The relationship between CDKN2A mutation and expression level of cuproptosis‐related genes in HCC. [file CNR2-6-e1904-s007.tif]

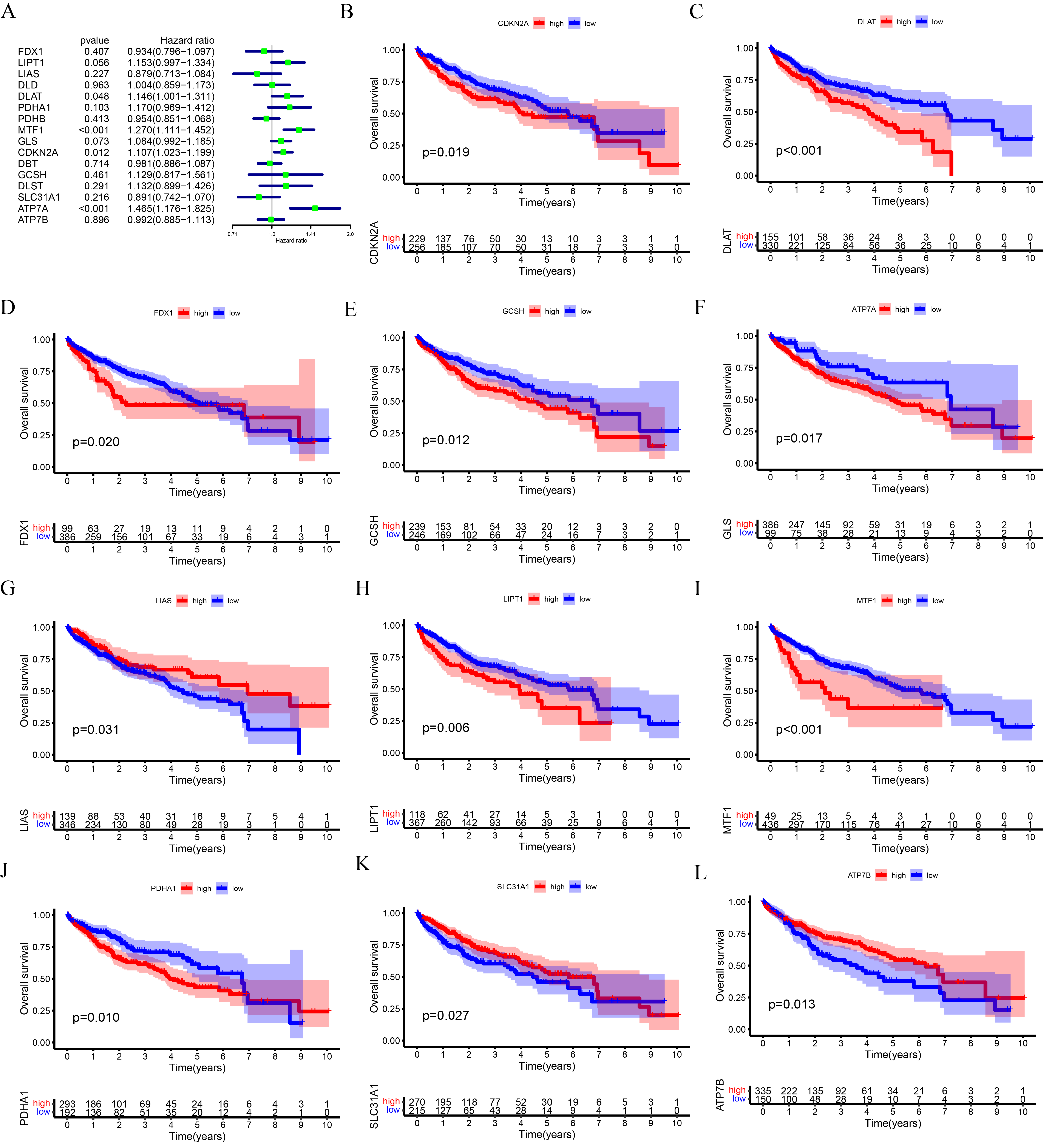

Supplement: Supplementary file 3 — Figure S3. A. The prognostic analyses for 16 CRGs in the two HCC cohorts using a univariate Cox regression model. Hazard ratio (>1) represented risk factors for survival and hazard ratio (<1) represented protective factors for survival. Survival analyses for low and high CRGs expression patient groups using Kaplan–Meier curves by Log‐rank test. B (CDKN2A, P = .019), C (DLAT, P < .001), D (FDX1, P = .020), E (GCSH, P = .012), F (ATP7A, P = 0.017), G (LIAS, P = .031), H (LIPT1, P = .006), I (MTF1, P = .001), J (PDHA1, P = .010), K (SLC31A1, P = .027), L (ATP7B, P = .013). [file CNR2-6-e1904-s006.tif]

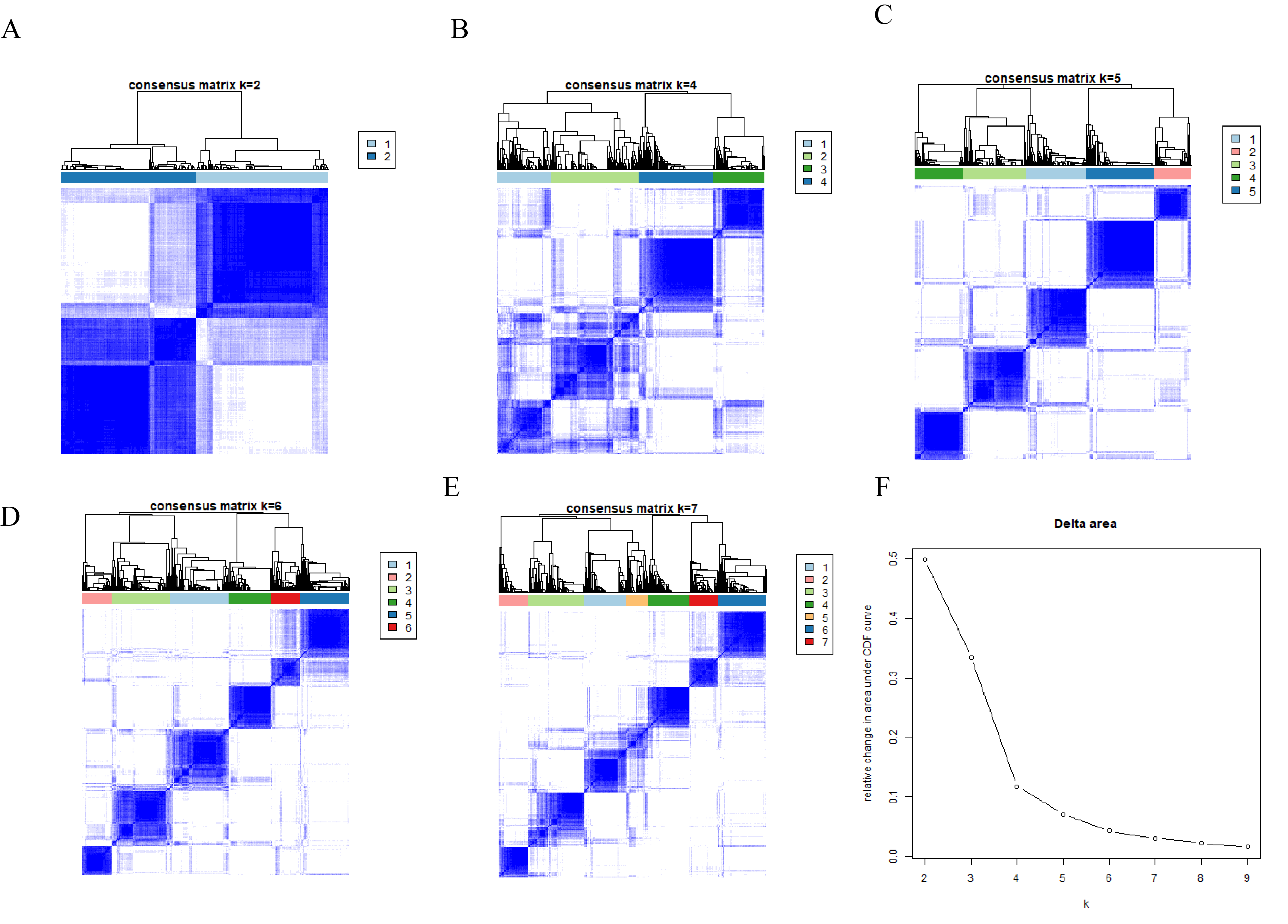

Supplement: Supplementary file 4 — Figure S4. Unsupervised clustering of CRGs and Consensus matrix heatmaps for K (K = 2, 4, 5, 6, 7). [file CNR2-6-e1904-s002.tif]

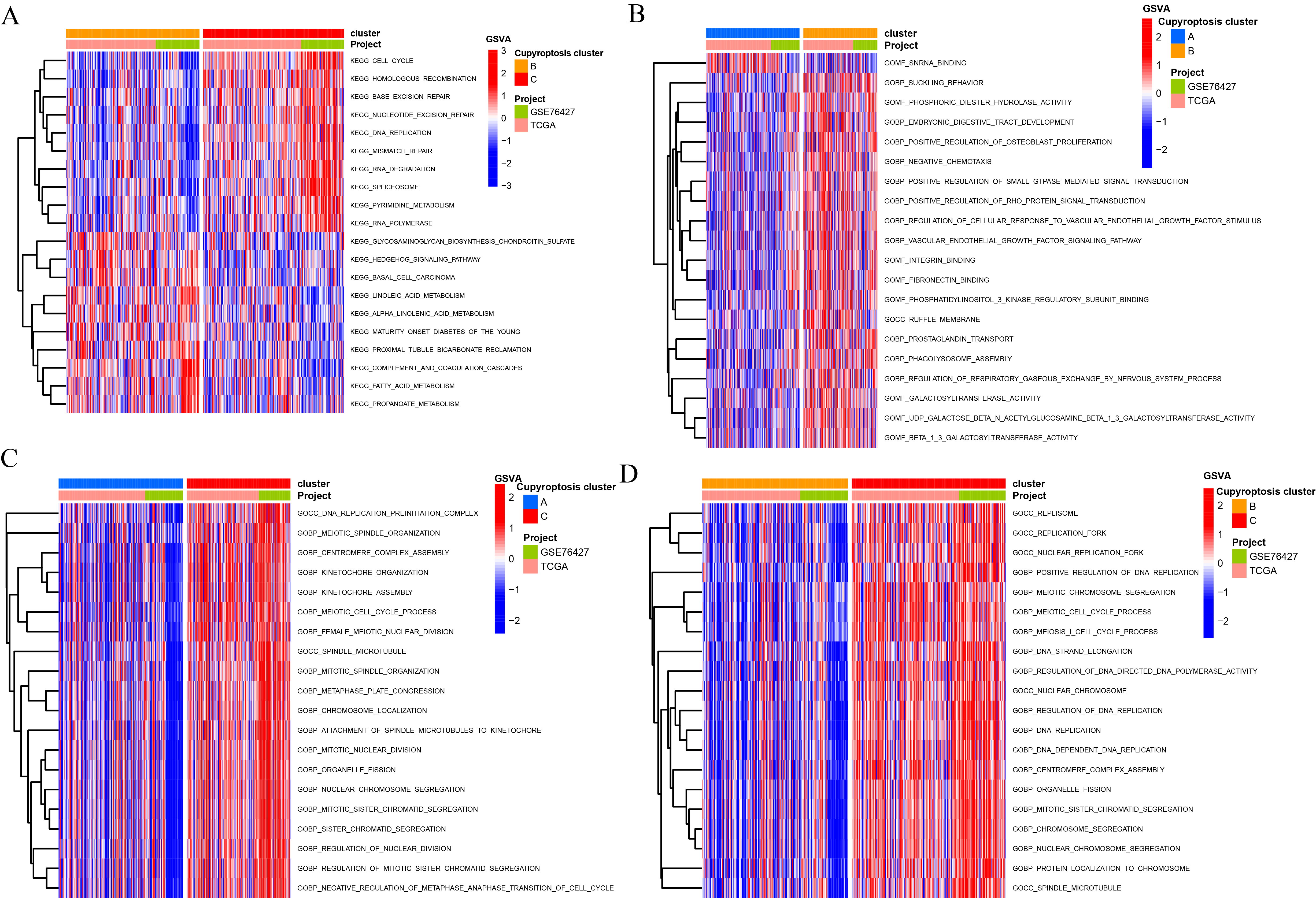

Supplement: Supplementary file 5 — Figure S5. GSVA enrichment analysis of biological pathways between three distinct subtypes by KEGG (A, cluster A vs. cluster C) and Go (B cluster A vs. cluster B, C cluster A vs. cluster C, C cluster B vs. cluster C) analyses. [file CNR2-6-e1904-s005.tif]

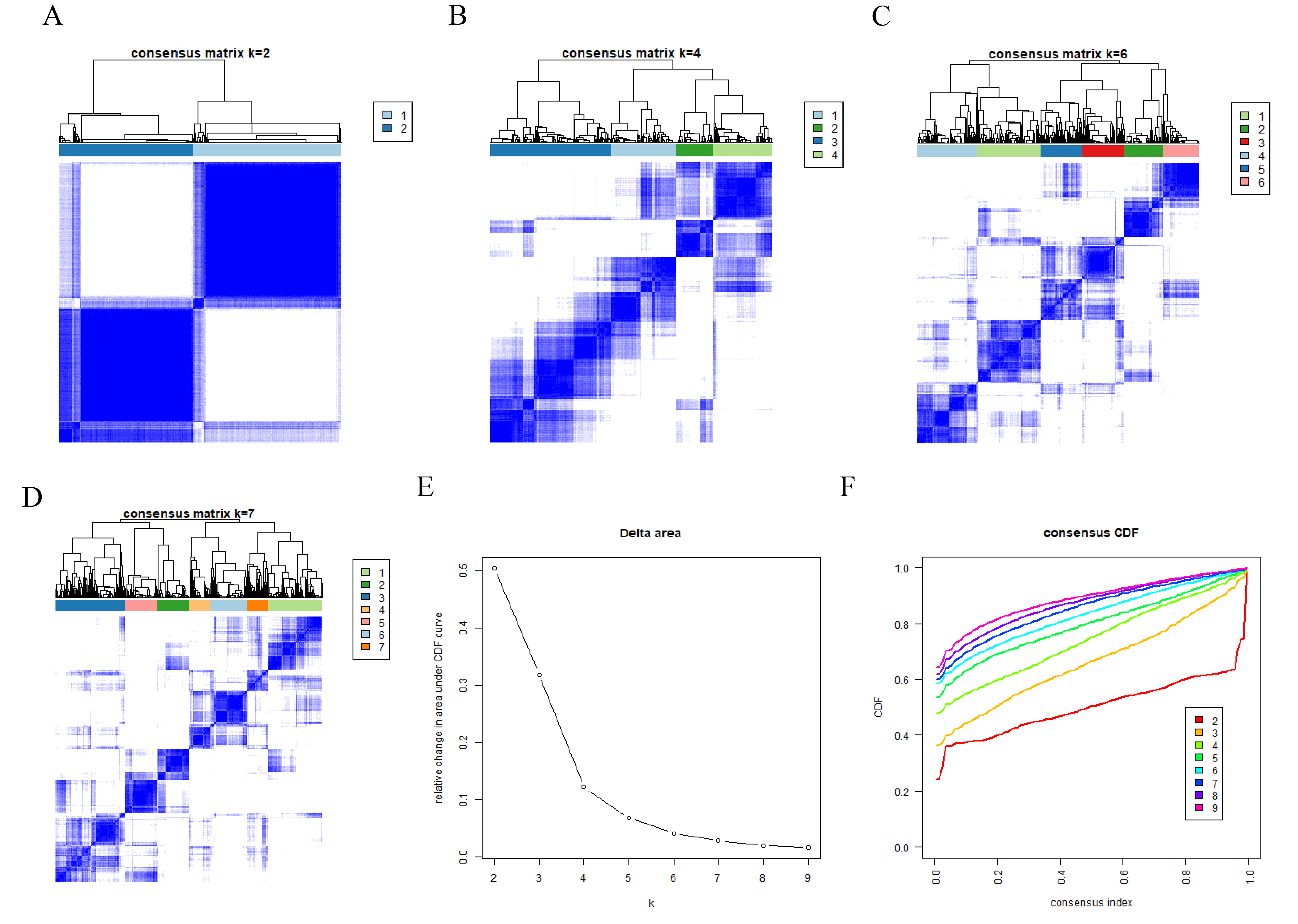

Supplement: Supplementary file 6 — Figure S6. Identification of gene subtypes based on DEGs among three cuproptosis subtypes in HCC cohort. [file CNR2-6-e1904-s003.tif]

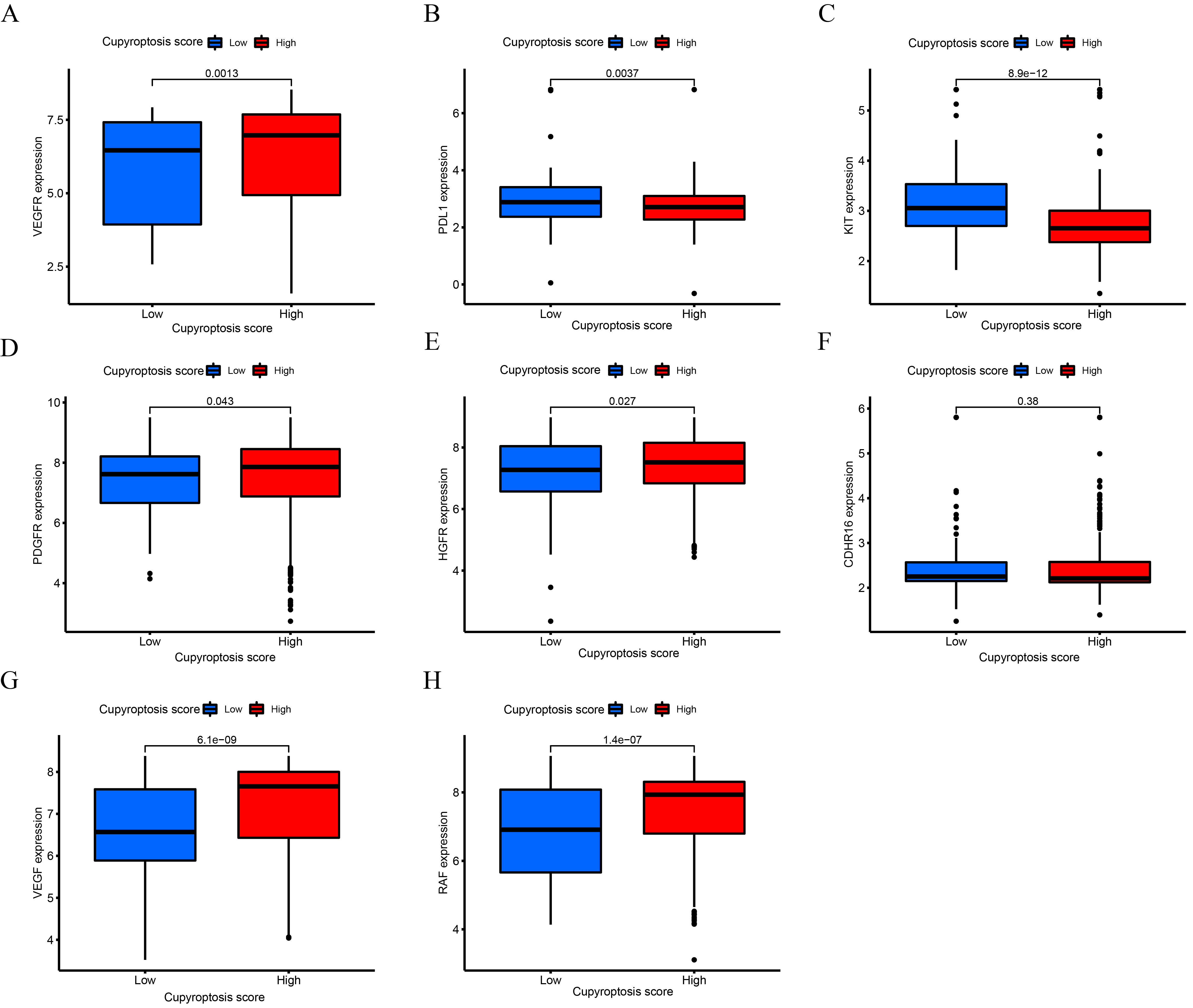

Supplement: Supplementary file 7 — Figure S7. Differences in immunotherapy target expression between low and high cuproptosis score groups in HCC. A (VECFR, P = .0013), B (PDL1, P = .0037), C (KIT, P < .001), D (PDGFR, P = .043), E (HGFR, P = .027), F (CDHR16, P = .38), G (VEGF, P < .001), H (RAF, P < .001). [file CNR2-6-e1904-s001.tif]
